# Thermodynamic analysis of an entropically driven, high-affinity nanobody-HIV p24 interaction

**DOI:** 10.1016/j.bpj.2022.12.019

**Published:** 2022-12-16

**Authors:** Jennifer C. Brookes, Eleanor R. Gray, Colleen N. Loynachan, Michelle J. Gut, Benjamin S. Miller, Alex P.S. Brogan, Rachel A. McKendry

**Affiliations:** 1 London Centre for Nanotechnology, Faculty of Maths and Physical Sciences, University College London, London, United Kingdom; 2 Department of Materials, Department of Bioengineering, and Institute of Biomedical Engineering, Imperial College London, London, United Kingdom; 3 Department of Chemistry, King’s College London, London, United Kingdom; 4 London Centre for Nanotechnology, Division of Medicine and Faculty of Maths and Physical Sciences, University College London, London, United Kingdom

## Abstract

Protein-protein interactions are fundamental to life processes. Complementary computational, structural, and biophysical studies of these interactions enable the forces behind their specificity and strength to be understood. Antibody fragments such as single-chain antibodies have the specificity and affinity of full antibodies but a fraction of their size, expediting whole molecule studies and distal effects without exceeding the computational capacity of modeling systems. We previously reported the crystal structure of a high-affinity nanobody 59H10 bound to HIV-1 capsid protein p24 and deduced key interactions using all-atom molecular dynamics simulations. We studied the properties of closely related medium (37E7) and low (48G11) affinity nanobodies, to understand how changes of three (37E7) or one (48G11) amino acids impacted these interactions; however, the contributions of enthalpy and entropy were not quantified. Here, we report the use of qualitative and quantitative experimental and in silico approaches to separate the contributions of enthalpy and entropy. We used complementary circular dichroism spectroscopy and molecular dynamics simulations to qualitatively delineate changes between nanobodies in isolation and complexed with p24. Using quantitative techniques such as isothermal titration calorimetry alongside WaterMap and Free Energy Perturbation protocols, we found the difference between high (59H10) and medium (37E7) affinity nanobodies on binding to HIV-1 p24 is entropically driven, accounted for by the release of unstable waters from the hydrophobic surface of 59H10. Our results provide an exemplar of the utility of parallel in vitro and in silico studies and highlight that differences in entropic interactions between amino acids and water molecules are sufficient to drive orders of magnitude differences in affinity.

## Significance

Proteins are important biomolecules in many life processes. Herein we analyze three llama antibody fragments (nanobodies) that bind p24, a structural HIV protein. One nanobody binds with high affinity, another (three amino acids different) binds moderately, and the third (one amino acid different) has completely abolished binding. Using atomistic modeling, we investigate individual interactions of amino acids and their bonds, compare bound and unbound forms of each nanobody, and highlight the importance of water in network formation around a key residue. Understanding processes like these aids exploitation of protein-protein interactions, which are critical to improving diagnostics and therapeutics of infectious diseases. Complementary computer and laboratory work generate molecular insights more powerful in combination than by either approach alone.

## Introduction

Interactions between antibodies and their targets are fundamental to areas as diverse as research in immunology to exploitation in industry in diagnostics and therapeutics. Nanobodies are the antigen-binding fragments of heavy-chain only antibodies, derived from camelids with no constant domains. They are typically 12–15 kDa in size and can be recombinantly produced from plasmids in *E. coli* or other protein production systems. Due to their small size and ease of manipulation, nanobodies are useful tools for exploring protein-protein interactions (1,2,3,4). Wider potential applications are also being exploited, for example, in therapeutics for cancers and neurodegenerative disorders, and as part of diagnostic tools including against SARS-CoV-2 (5,6,7,8).

Protein binding can be considered in terms of changes in enthalpy (ΔH) and entropy (ΔS) when the two individual components come together to form a complex, potentially with associated changes in structure. Complexation will only occur if the overall thermodynamic potential, the Gibbs free energy (ΔG), is negative. ΔG is a function of entropy and enthalpy at a given temperature and pressure (ΔG = ΔH – TΔS), and for biological molecules, it can also be calculated from the dissociation constant (*K*_D_) using the equation ΔG = –RTln(1/*K*_D_). Protein binding affinity may be weak (with a dissociation constant, *K*_*D*_, of 10^−6^ M) or high (*K*_D_ of 10^−9^ M).

**Figure 1:**
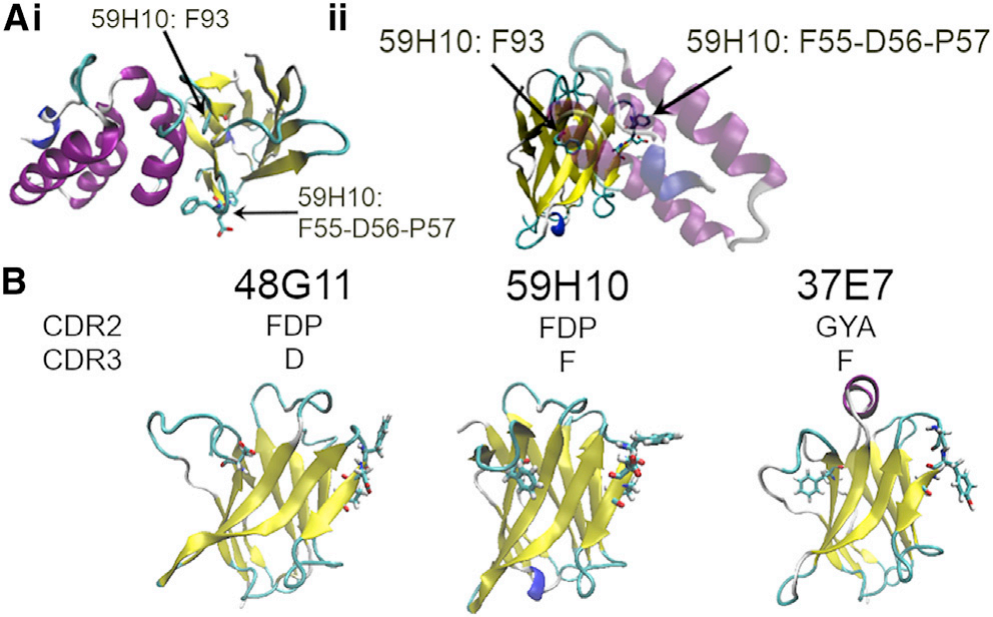
Structural and genetic data of p24 and the nanobodies considered in this study. (*a*) Two views of the complex formed by 59H10 (*yellow*) and HIV-1 p24 (*purple*). Amino acids that differ between 59H10, 48G11, and 37E7 are highlighted. *i*) View from above 59H10 CDR loops. *ii*) Side view. (*b*) Sequences of 59H10, 48G11, and 37E7, where variant, and comparison of the structures of the three nanobodies showing secondary structure (β-sheets in *yellow*, α-helices in *purple*, and unstructured loops in *blue*). To highlight the differences, the variant amino acids are rendered in licorice. For full sequences, refer to Gray et al. (9). To see this figure in color, go online.

Recently, we reported the structure of a nanobody, 59H10, that binds to the capsid protein of HIV-1, p24 (9) (Fig. 1). 59H10 was originally isolated from a nanobody panel isolated after inoculation of *Lama glama* with HIV-1 p24, and it binds to p24 from a broad range of different HIV-1 subtypes at high affinity (*K*_*D*_ < 1nM). The HIV-1 p24 capsid protein is present in about 1500 copies per virus, and it is used as a biomarker for early-stage detection of HIV infection (10,11). It is a predominantly α-helical protein formed of 11 helices within two subdomains, joined by a flexible linker region between helices 7 and 8 (12). 59H10 binds to the C-terminal end, to helices 10 and 11 with contributions to binding from 59H10 complementarity determining regions (CDR) 1, 2, and 3 and the β-sheet (9,12). The interaction is mediated by a salt bridge between 59H10 R50 and p24 E213, hydrogen bonds, and the matching of hydrophobic patches (59H10 F93 and p24 A204-L205) (9) (Fig. S1). In a nonbinding artificial variant of 59H10 named 48G11, this hydrophobic patch is perturbed by the single site mutation of F93D, abolishing measurable binding. In an intermediate-binding natural variant 37E7 (isolated from the same library as 59H10), CDR2 is modified (59H10 FDP to 37E7 GYA) with ensuing effects on the CDR1 and cascade effects of charge distribution over the whole binding façade (9).

Previously we studied the interaction between 59H10 and p24 using endpoint binding ELISA, crystallography, molecular dynamics (MD), and biolayer interferometry (9). Herein we report details of a study that maximizes the interplay of wet-lab biophysical and in silico characterization of the thermodynamic parameters of binding of these nanobodies to HIV-1 p24. We complement our previous crystal structure with circular dichroism (CD) spectroscopy of nanobodies bound and unbound to p24, determine the enthalpic and entropic contributions to binding using isothermal titration calorimetry (ITC), and compare with inhomogeneous fluid solvation theory implemented by *WaterMap* from the Schrodinger Suite (13,14). WaterMap determines the solvent enthalpy and entropy at the nanobody binding interface. We have used this to qualify and quantify the high, moderate, and minimal binding affinity of the three nanobodies, to complement the ITC experiments and interpret affinities at an atomic level. Mapping the thermodynamics of waters with explicit solvent simulations in this way aids understanding of the driving forces of this high-affinity interaction. Furthermore, free energy perturbation (FEP) is applied to the nanobody proteins to understand the effect of the mutations, using the Schrodinger FEP+ protocol (15). Although the parameters described here apply to the binding of 59H10, 48G11, and 37E7 to p24, the principles uncovered and the experimental methods are applicable to understanding any antibody-antigen pair. The interplay and intersection of experimental and computational studies allows a greater range of hypotheses to be tested at greater depth than either discipline alone (16).

## Materials and methods

### Recombinant p24 protein

Purified p24 was generated in-house using a template from pWISP98-85 (NIH ARP) and the following primers to add His_6_-Cys to the N-terminal end.

EG298 GGTTTCCCTCTAGAAATAATTTTGTTTAACTTTAAGAAGGAGATATGCATCATCATCATCATCATTGCCCGATCGTGCAGAACC

EG299 GGCTTTGTTAGCAGCCGGATCCTTACAAAACTCTTGCTTTATGG

The PCR fragment and pET3a plasmid were digested with XbaI and BamHI (sites underlined) and ligated to generate the pET3a-His-p24 plasmid, which was verified by sequencing and transformed into BL21-Star(DE3)pLysS for protein production. Production from *E. coli* cultures was performed as described previously (33). Proteins were stored in 10 mM phosphate buffer (pH 8.0) with 25 mM NaCl (“storage buffer”).

### Molecular dynamics simulations

For the 59H10-p24 complex (PDB: 5O2U) was downloaded and prepared using Maestro’s Protein Preparation Wizard (Schrödinger Release 2019-4). The N- and C-termini were capped, hydrogen atoms were added, and ionization states of the residues were determined at pH 7.0 by Propka. To create the mutations in the nanobodies for 37E7 and 48G11 models, individual residues were mutated from the structure of the prepared 59H10 nanobody as a template. A rotamer library derived from high-resolution x-ray structures was used to select conformations of the mutations. Then all three nanobodies created for MD (in isolation and in complexes, deleting or retaining the p24 respectively for *apo* and *holo* forms) were minimized with initial restraints on the heavy atoms and then equilibrated. Consequent MD and the analysis presented here is from 1000-ns-long production runs at 300 K for (1) nanobodies in isolation and (2) nanobodies in complex with p24 antigen, in each case surrounded by a cubic water box, using SPC explicit solvent. All MD, WaterMap, and FEP+ calculations were run with Desmond (34,35) and using the OPLS3e force field (36,37).

### WaterMap

After this initial preparation (as in previous section), proteins were placed in an orthorhombic box with explicit solvent TIP4P, equilibrated, and then simulated for 2 ns duration in the NPT ensemble at 300 K and 1 atm using the OPLS3e force field for the proteins. The values calculated in WaterMap correspond to the average excess enthalpy, entropy and free energy that the hydration site (water molecule, “clusters” from the MD analysis) would have relative to bulk water. Calculations were performed with (holo) and without (apo) the bound p24 protein, and the regions probed were within 10 Å of the protein-protein binding interfaces (usually this is defined by a small ligand binding site, in this case the whole binding surface of the nanobodies were taken as the binding site).

### FEP+

FEP+ calculations followed on from the initial preparation of PDB: 5O2U as above, with the relevant mutations identified on the nanobody 59H10 to create 37E7 and 48G11. The mutated residues fall within the replica exchange solute tempering (REST) region (15), and replicas are exchanged every 1.2 ps. An initial relaxation protocol was performed, and a grand canonical Monte Carlo water sampling period throughout the equilibration and production REST run. This protocol is referred to as μVT (26), maintained at a temperature 300 K. A cubic box and solvation buffer of 8 Å and 0.15 M NaCl was used for charge altering mutations, and in this case, each perturbation was performed over 24 λ windows. For noncharge altering mutations a buffer of 5 Å is used and 12 λ windows. 16 λ windows are used for core-hopping transformations (such as to or from proline) (15). Each lambda window was simulated for 25 ns, and the SPC water model was used.

### Isothermal titration calorimetry

A PEAQ-ITC (Malvern Panalytical, UK) in the Department of Anatomy at UCL was used for direct measurement of protein binding energetics of nanobody-p24 complex formation according to standard methodology, adapted to the expected high affinity (38,39). All experiments were conducted in protein storage buffer (10 mM phosphate, pH 8, with 25 mM NaCl). All experiments and controls were performed in at least triplicate with the following parameters: syringe, 200 μM nanobody; sample cell, 12.5 μM His-p24; reference cell, 18.2 MΩ water; temperature, 25.1°C; reference power, 10; feedback, high; stir speed, 750 rpm; initial delay time, 120 s; injections, 19 total (1 × 0.4 μL, 18 × 2.0 μL); injection spacing, 150 s; injection duration, 4 s. For control experiments (to measure heat of dilution), titrant or sample was replaced by storage buffer. Before every experiment the cleanliness and the stability of the system were checked by titrating water into water using the same settings as above, except reference power was set to 5 and injection spacing to 120 s. In between experiments, the sample cell and syringe were cleaned.

### Circular dichroism spectroscopy

CD spectroscopy was performed using a Chirascan (Applied Photophysics, UK) at the Macromolecular Interactions Facility at Imperial College, London. Spectra were collected between 260 nm and 190 nm with an integration time of 4 s and a data interval of 1 nm at 25°C. Nanobodies 59H10, 48G11, or 37E7 and His-p24 in storage buffer were run in 1-mm quartz cuvettes, with a typical protein concentration of 0.2 mg/mL. His-p24 sample was also spiked with 59H10 in a molar ratio of ∼1:1 (0.1 mg/mL 59H10) and ∼1:2 (0.2 mg/mL 59H10). To analyze the binding spectra of the 59H10-His-p24 interaction, the signal of the single protein spectra was summated and compared with the measured spectra of the interaction. To analyze the CD spectra, absorbance values were converted into mean residue ellipticity using the following equation:

$$\theta_{mre}=\frac{MW\cdot \theta }{10\cdot (N-1)\cdot l\cdot c}\text{,}$$

where θ_mre_ = mean residue ellipticity [deg·cm^2^·dmol^−1^], MW = molar weight [g·mol^−1^], θ = ellipticity [deg], N = number of residues in the protein (N – 1 number of peptide bonds), l = pathlength [cm], and c = concentration [g·cm^−3^] (20).

## Results

**Figure 2:**
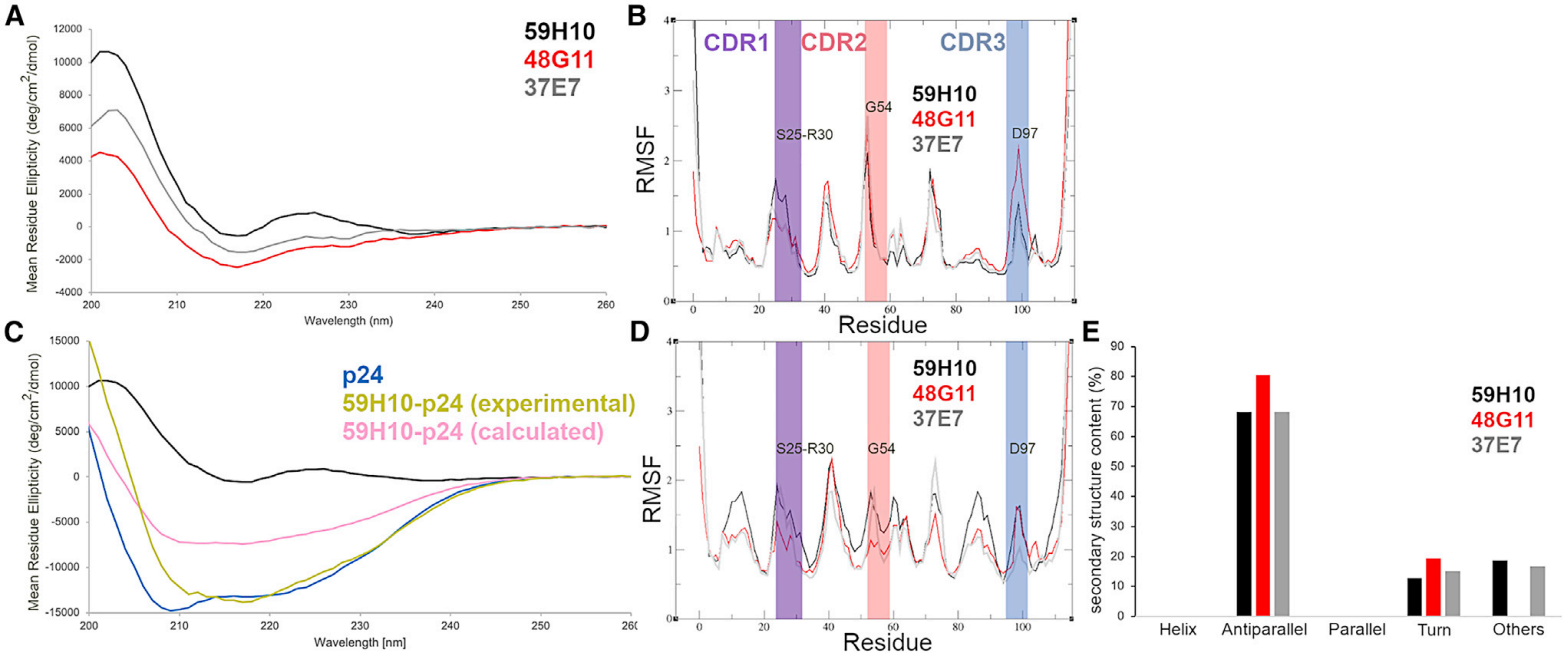
Effect of variations in CDR2 and CDR3 explored through circular dichroism and molecular dynamics simulations of each nanobody. (*a*) CD spectroscopy of the three nanobody variants. (*b*) Map of the flexibility of residues, with RMSF of the Cα carbon on each residue versus residue number for nanobody structure in isolation with CDR1 (*purple band*), CDR2 (*red band*), and CDR3 (*blue band*) regions denoted. (*c*) CD spectroscopy of p24 and 59H10, individually and as a bound pair. (*d*) Map of the flexibility of residues, with RMSF of the Cα carbon on each residue versus residue number for the nanobodies in complex with p24 with CDR1 (*purple band*), CDR2 (*red band*), and CDR3 (*blue band*) regions denoted. (*e*) The online tool BeStSel was used to analyze the relative proportions of the different types of secondary structure. Although 59H10 and 37E7 appear relatively similar in the proportion of antiparallel β-sheets, turns, and unclassified structures (others such as π-helices, β-bridges, and disordered loops), 48G11 is qualitatively distinct. To see this figure in color, go online.

Initial investigations examined the nanobodies as individual structures by in vitro and in silico methods, independent of any binding partners. CD spectroscopy was used to assess the relative levels of secondary structure of 59H10 (strong binding to p24), 48G11 (no binding to p24), and 37E7 (medium binding to p24) (Fig. 2 *a*). The derived mean residue ellipticity graphs show that the structures comprise predominantly antiparallel β-sheets for all three nanobodies, as would be expected (17),(18). However, 59H10 was the only nanobody to generate a positive ellipticity peak centered on 225 nm, indicating the potential presence of a type II (polyproline) helix, which is frequently found in protein-protein interactions in bound proteins (PPI) (19). The absence of this peak in 48G11 and 37E7 could indicate that with a higher underlying level of disorder in these nanobodies, they may be missing this structural feature. Potentially disordered regions such as these may give rise to more unfavorable entropy upon complexation, as greater structural shifts are required and are more energetically costly compared with a region that already has associated secondary structure. MD simulations were run for 1000 ns for the three nanobodies in isolation to assess movement of residues. Fig. 2 *b* shows the resulting root mean-square fluctuation (RMSF) of the individual amino acids, with respect to the average structure, over this time. RMSF analysis enables consideration of the flexibility of individual residues, where at room temperature a low RMSF of <1.9 Å indicates residues with a structure close to the input crystal coordinates or average of the simulation. Fluctuations higher than this often point to the less-structured and highly fluctuating loop regions (versus α helices and β-sheets). This allows qualitative comparison of the nanobodies rather than quantitative assessment of entropic binding costs, but comparisons and differences in residues across a data set can point to residues possibly responsible for differences in affinity. Highlighted are the three main areas with differences in the RMSF between the three nanobodies (excluding N- and C-termini), which fall in the CDR1 (purple), CDR2 (red), and CDR3 (blue) regions. Amino acids S25–R30 of 59H10 (within CDR1) have higher RMSF peaks, than 48G11 and 37E7, and these could correspond to the potential type II (polyproline) helix, seen from the CD data (Fig. 2 *a*). Also notably high in RMSF are G54 of CDR2 (found at the top edge of the binding interface) and D97 of CDR3 (also at the top edge of the binding interface), which are understandably fluctuating more in all three nanobodies in isolation rather than when in complexation. The dominant D97 fluctuation of 48G11 (in isolation in Fig. 2 *b*) is likely because there is no internal salt bridge between D97 and R50, as it is disturbed by the F93D mutation, allowing the D97 to fluctuate much more in solution than if it was held in a more favorable electrostatic network forming intraprotein contacts.

We next investigated interactions between the nanobodies and their binding partner: p24. Since CD spectra are additive, a mixture of two pure proteins can be analyzed and compared with the individual protein spectra (20). If the proteins do not interact and do not form a complex, or no concomitant structural change occurs when they interact, the summated single spectra (“calculated”) and the spectra of the mixture (“experimental”) should overlay. The spectra of p24 alone (Fig. 2 *c*, blue line) showed a typical α-helical structure, concordant with previous analyses (21,22,23). The spectrum from the equimolar mixture of 59H10 and p24 exhibited moderate differences in the CD spectra compared with the summed single protein spectra of the components (Fig. 2 *c*, green versus pink lines, respectively). The tool BeStSel (http://bestsel.elte.hu (24)) was used to assess the relative proportions of secondary structure features through analysis of the CD data, shown in Fig. 2 *e* and Fig. S2. The dominant feature of the nanobody structures when in isolation are antiparallel β-sheets. 48G11 contains the largest proportion of “turns,” which may include 3_10_ -helices. A comparison of the BeStSel analyses of the summated 59H10 and p24 CD spectra and experimental data for the 59H10-p24 complex indicate an approximately 10% reduction in α-helices (p24) and antiparallel β-sheets (59H10) in the experimental data compared with that expected from the summated spectra (Fig. S2). An increase is seen in the class of “other,” corresponding to π-helices, β-bridges, and disordered loops. Loop transitions to turns can be observed in the MD results as 3_10_ -helices form between residues D61 and K64 for 37E7 and 48G11, both in isolation and in complex, but they are not seen for 59H10. This indicates that minor changes in secondary structure occur when 59H10 binds to p24. To assess how these changes correspond to structural movements during binding, we used MD to analyze the RMSF of the nanobody-p24 complex over a 1000-ns MD simulation. The fluctuations of the nanobody in complex are presented in Fig. 2 *d* and can be contrasted with Fig. 2 *b*. CDR regions of the nanobodies fluctuate considerably less when in complex with p24, and the lower loop regions (at the bottom of the PPI) are comparatively more mobile. Although the CDR1 does not undergo much movement in complex or in isolation (under 2Å RMSF), G54 of CDR2 and D97 of CDR3, which lie at the top edges of the protein-protein binding interface, fluctuate much less while constrained in complex compared with in isolation. Overall, our MD analyses indicate that there are no large secondary structural changes (from α-helix to β-sheet or vice versa, or significant loss of either to unstructured forms) to the nanobody upon complexation. Analysis of all three complexes, even 48G11-p24, shows that when the nanobody and antigen are bound, they are stable (the binding event itself is not computationally tractable). This is seen in the RMSF and secondary structure analysis (described above), but also in the RMSD, radius of gyration, the kinetic and potential energy, temperature, and volume plots (over time). Important to note also is that these 1-*μ*s simulations are a very useful precursor to running WaterMap and FEP+. These long-scale unbiased all-atom MD establish that the complexes remain bound, and indicate they are a realistic “snapshot” of interactions that can be used as input in analysis of water and free energy simulations. Showing that the binding mode remains similar over time (as is demonstrated in a stable RMSD, for example) provides support for the applicability of the methods described below.

**Figure 3:**
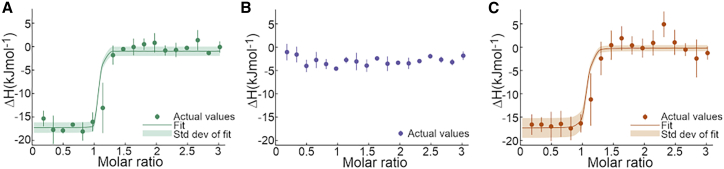
Analysis of 59H10, 48G11, and 37E7 interaction with p24. Shown is the change in enthalpy as the molar ratio of reagents alters for (*a*) 59H10 and p24, (*b*) 48G11 and p24, and (*c*) 37E7 and p24. Graphs show combined mean results from at least three experiments performed per reagent set; error bars show standard deviations. Raw thermograms are shown in Fig. S3. Control titrations are shown in Fig. S4. To see this figure in color, go online.

To measure the enthalpy of the binding interaction between the nanobodies and p24, ITC experiments were performed, titrating the nanobody into p24. The normalized injection heat and fitted isotherms are shown in Fig. 3 *a*–*c* for 59H10, 48G11, and 37E7, respectively. The derived enthalpy of the reactions is given in Table 1. Raw thermograms from which data in Fig. 3 are derived are shown in Fig. S3. Control experiments to measure the heats of dilution of all nanobodies and p24 are shown in Fig. S4.

**Table 1:** Thermodynamic parameters of 59H10 and 37E7 binding to p24

Nanobody	K_D_ (nM)	ΔG in kcal mol^−1^ (kJ mol^−1^)	ΔH in kcal mol^−1^ (kJ mol^−1^)	–TΔS in kcal mol^−1^ (kJ mol^−1^)	ΔS in cal mol^−1^ K^−1^ (J mol^−1^ K^−1^)
59H10	0.77	−12.5 (−52.1)	−3.9 (−16.3) ± 0.4 (1.5)	−8.6 (−35.8) ± 0.2 (1.0)	28.7 (120)
48G11 (F93D)	NC	NC	NC	NC	NC
37E7 (F55G-D56Y-P57A)	9.6	−10.9 (−45.8)	−4.1 (−17.1) ± 0.4 (1.6)	−6.9 (−28.7) ± 0.4 (1.5)	23 (96)

Gibbs free energy is derived from the equation ΔG = –RTln(1/K_D_) using affinity constants (measured K_D_) from biolayer interferometry experiments performed in the same nanobody storage buffer (Fig. S5). Enthalpies are derived from the ITC experiments shown in Fig. 3, and entropy calculated from the equation ΔG = ΔH – TΔS. T for the ITC experiments was 25.1°C. NC, not calculable.

The results shown in Fig. 3 and Table 1 indicate that the enthalpy change in the highly favorable interaction of 59H10 with p24 is not significantly different to the enthalpy change in the moderately favorable interaction of 37E7 and p24. When 48G11 interacts with p24, there is no measurable change in enthalpy that can be detected through ITC. The more favorable change in Gibbs free energy of p24 binding to 59H10 compared with 37E7 that drives the reaction is likely therefore entropically driven. As entropy-driven binding can be explained as a hydrophobic effect caused by the release of low-entropy immobilized water molecules (25), we hypothesized that this might be true for our nanobodies, comparing the hydrophobicity of the nanobody-p24 interfaces. Although the CD results suggest that a polyproline helix formed in 59H10 could account for some differences in binding, neither CD nor MD results (Fig. 2) indicate a large conformational change that would obviously account for this entropic effect. However, the possibility should not be completely discounted that such a structural feature (or lack of) could contribute to local protein mobility and disorder and would be expensed at an entropic cost upon complexation with p24. Regardless of this possibility, the indication of entropic gain due to desolvation when these nanobodies form such complexes appeared to be particularly relevant to this contrasting set of three affinities (high, moderate, and none). This was indicated largely given the striking result of effectively abolishing binding with the swapping of just one hydrophobic residue (59H10) with a charged one (48G11). It can reasonably be assumed the incurring entropic difference is more likely due to the impact on waters at this binding site, rather than any resulting large structural conformational entropy change due to one amino acid change. To understand what this means at the atomistic level, WaterMap calculations can be used to quantify the solvent enthalpy and entropy contributions of waters at the protein-protein interfaces.

WaterMap employs inhomogeneous fluid solvation theory to determine the free energy of water molecules around the protein relative to bulk water molecules. After a 2-ns MD simulation, with heavy restraints on the protein atoms, the position of every water in the region of interest is clustered and analyzed. Locations of high water density are labeled “hydration sites.” The enthalpy contribution of each site is calculated from the average nonbonded interaction energy of each water and the rest of the system over the trajectory. In contrast, the entropy is found from the numerical integration of a local expansion of spatial and orientational correlation functions (13). Assigning “hydration spheres” allows analysis of populated sites from the simulation, with associated thermodynamic properties, locations, and directionality. There is also a way to analyze the free energy as a continuous grid and create maps, similar to electrostatic potential plots, as is also shown in these figures with surface isovalues of 0.033 kcal mol^−1^Ȧ^−3^. Analyzing the distribution of waters with WaterMap like this is more typically used to aid drug design, where the mapping can highlight areas of the molecule to design that would displace unfavorable waters (for example, and so aid design of high affinity and improved selectivity of small molecules). The nanobodies investigated here offer an interesting and less typical example of intermolecular interaction exploration and demonstrate the power and influence of waters in PPIs. This protocol thus demonstrates great potential for therapeutic nanobody and antibody design analogous to the great success WaterMap (and FEP+) has already evidenced in small molecule drug design.

**Figure 4:**
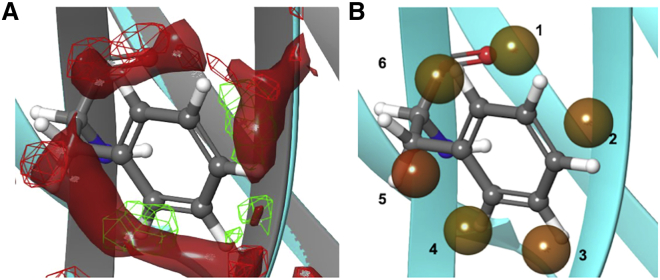
WaterMaps of apo nanobody (no antigen bound) 59H10 versus 48G11. Comparing free energy density, a continuous water map, with a surface isovalue of 0.033 kcal mol^−1^Ȧ^−3^ of the free energy of the water molecules at the nanobody interface (*a*) 59H10 F93 (*blue*, high-affinity, solid isosurface) and 48G11 D93 (*gray*, binding abolished, mesh isosurface) zoomed into the residue site and phenylalanine shown in ball and stick. The energy distribution surrounding the phenylalanine in 59H10 demonstrates these waters would be favorably displaced by a hydrophobic interface (the p24 antigen), as doing so releases ∼6.6 kcal/mol –TΔS, indicating that this nanobody-antigen PPI is entropically driven for 59H10. (*b*) Shows the hydration sites (*spheres*) for waters surrounding the phenylalanine (in 59H10, *blue*) for those ΔG > |2| kcal/mol (colored in relative; *red* is unfavorable, *green* is favorable). To see this figure in color, go online.

Fig. 4 demonstrates a close-up view of the WaterMaps to enable comparison of 59H10 (solid isosurface) and 48G11 (mesh isosurface) at the F93D mutation site. F93 of 59H10 is strikingly circled by unfavorable free energy density, which is by contrast disrupted with the D93 of 48G11, wherein favorable free energy density can be observed in proximity to the charged aspartic acid. This free energy density maps out a ring directly around the F93 in 59H10, which upon further analysis, reveals itself to be similar and “ice-like,” as in the extremely high-affinity streptavidin-biotin interaction, where a penalty of such a ring of waters forming for the proteins in isolation is stabilized by binding and accounts for the physical basis of this “super” affinity (14). Fig. 4 *b* shows plots of the hydration sites as spheres, for all those whereby ΔG > |2| kcal mol^−1^ (i.e., the most unfavorable/favorable). The unfavorable sites that encircle the F93 are labeled 1–6. The thermodynamic contributions of these 1-6 are listed in Table 2. Note the circling waters are all unstable and unfavorable in every component: for the free energy, enthalpy, and entropy (waters in association with a protein always have an entropic penalty). Comparing the WaterMaps at this mutation site show there is a clear difference in density surrounding F93 and D93 illustrating whether waters are held at this site unfavorably (59H10) or favorably (48G11). This can guide understanding of the stark difference in the entropically driven interaction for 59H10 versus no measurable interaction for 48G11 (noncalculable, Table 1). It can be hypothesized from Fig. 4 *a* that the hydrophobic face of 59H10 would favor the release of the unstable waters (red solid free energy density) in favor of a hydrophobic interaction, whereas 48G11 interacts more favorably with waters (green mesh free energy density), which are more stable and energetically quiescent in hydrating the aspartic acid and would much less readily interact with a hydrophobic face, reflected in the lack of binding to p24. The reading of a WaterMap is a qualitative result, but it can be quantified by the calculated change in ΔΔG (the difference in the change in binding free energy, in this case for the perturbation of F93 to D93, i.e., 59H10 versus 48G11) from the FEP+ calculation that predicts a ΔΔG = 7.84 ± 0.53 kcal mol^−1^ (32.8 ± 2.2 kJ mol^−1^). This is shown in Table 3, by 59H10 compared with 48G11. This is the energetic “cost” of mutation of F93D that equates to about five orders of magnitude decrease in binding affinity. The combination of WaterMap visual and FEP+ calculation support and explain the experimental result that the affinity is completely diminished on account of and by the mutation of F93D. It is a change that completely disrupts an advantageous hydrophobic effect of water displacement.

**Table 2:** Calculated values for the water sites depicted in Fig. 4

Site	Occupancy	ΔH kcalmol^−1^	–TΔS kcalmol^−1^	ΔG kcalmol^−1^	Hydrogen bonds (water to water)	Hydrogen bonds (protein to water)
1	0.29	2.03	0.81	2.84	2.01	0.00
2	0.35	2.30	0.95	3.25	1.96	0.00
3	0.52	1.86	1.59	3.45	1.65	0.55
4	0.44	1.41	1.34	2.75	1.72	0.44
5	0.37	3.22	1.08	4.30	1.80	0.00
6	0.32	2.13	0.83	2.96	2.19	0.00

Shown are the quantitative analysis including the number of hydrogen bonds between waters and between the protein and waters.

**Table 3:** FEP+ data for 25-ns simulations, using the μVT protocol (26), 300 K, and OPLS3e force field and SPC water model

Mutation	Predicted ΔΔG in kcal mol^−1^ (kJ mol^−1^)	Predicted ΔΔG Error in kcal mol^−1^ (kJ mol^−1^)
48G11 (F93D)	7.84 (32.8)	0.53 (2.2)
37E7 (F55G-D56Y-P57A)	3.08 (12.9)	0. 71 (3.0)
D56Y-P57A	0.67 (2.8)	0.74 (3.1)
F55G	3.75 (15.7)	0.52 (2.2)
F55G-D56Y	2.27 (9.5)	0.62 (2.6)
P57A	0.53 (2.2)	0.61 (2.55)
D56Y	−0.24 (−1.0)	0.61 (2.55)
F55G-P57A	4.03 (16.9)	0.62 (2.6)

Shown are the results for predicted relative energies and the associated convergence errors upon individual and combined mutations along the transformation from 59H10 to 48G11 (F93D) and 37E7 (F55G-D56Y-P57A) and also the individual mutations and their contributions that in combination effect the transition from 59H10 to 37E7.

**Figure 5:**
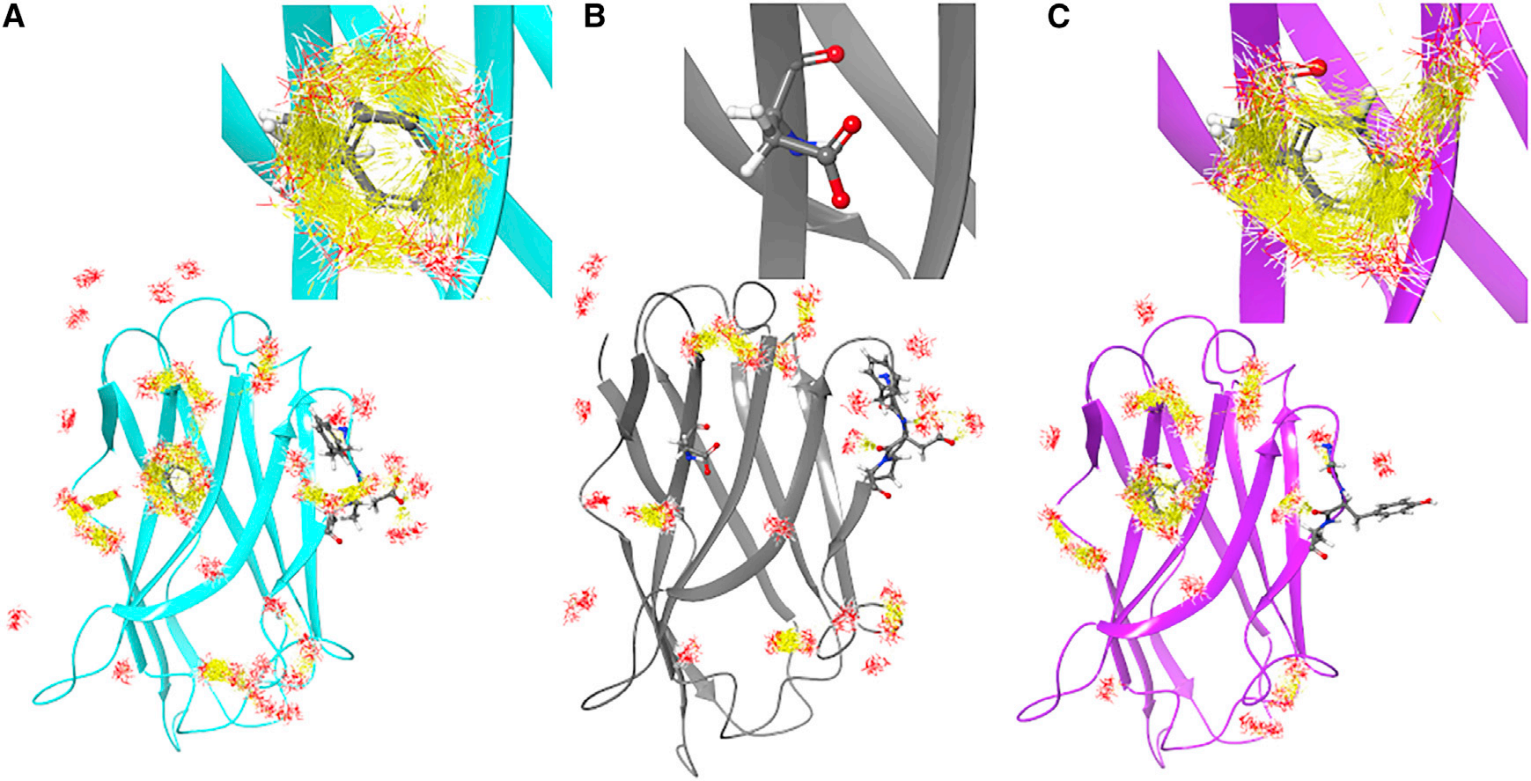
WaterMaps of apo nanobody (no antigen bound) full view all. Comparing the WaterMaps of (*a*) 59H10 (*in blue*) (*b*) 48G11 (*gray*), and (*c*) 37E7 (*purple*), for those hydration sites ΔG > |2| kcal/mol, with directional depiction of the waters. In yellow are shown the hydrogen bonds between those waters at the hydration sites over the trajectory analysis of the simulation. Inset are zooms to the F93D site. Note the “ice-like” ring around F93 of 59H10. To see this figure in color, go online.

Another way of depicting the importance of interacting waters is shown in Fig. 5, which displays the full protein binding face, and close ups inset, for all three nanobodies for comparison, but it depicts now the direction of waters taken from the simulation analysis and with rendering for hydrogen bonds displayed (in yellow); again only sites ΔG > |2| kcal mol^−1^ are shown. It is interesting to note that the highly unfavorable ring of waters around 59H10 F93 (with a hydrophobic side chain) largely interact with themselves through hydrogen bonds, as opposed to the protein surface, forming a crystal-like (“ice-like”) ring (14). This is noticeably clear for 59H10, less markedly for 37E7, and not at all for the nonbinder 48G11 that has hydrophilic D93 (with a negatively charged side chain). Note also the G55-Y56-A57 (37E7) area is also diminished in hydrogen bonds networks among water sites compared with F55-D56-P57 (59H10/48G11). This hydrogen bond rendering style demonstrates the “ice-like” ring phenomenon previously observed in the extremely high-affinity streptavidin-biotin interaction (14).

**Figure 6:**
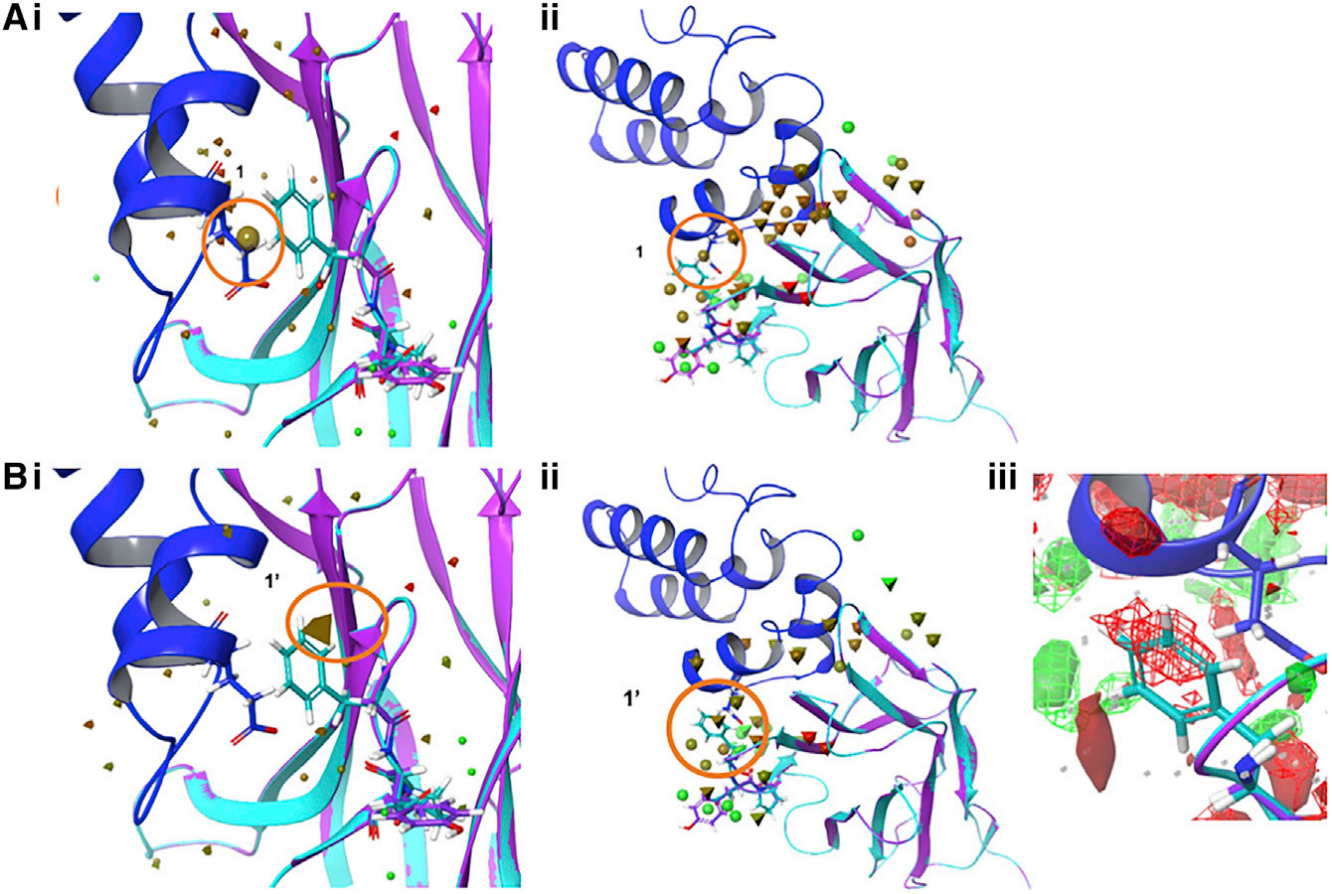
WaterMaps of apo and holo nanobodies (59H10 and 37E7). (*a*) Apo 59H10 and 37E7 (no antigen bound). *i*) Comparison of hydration sites of magnitude ΔG > |2| kcal/mol and 6 Å from the nanobody chain B for 59H10 (F55-D56-P57) in spheres and 37E7 (G55-Y56-A57) in pyramids (colored in relative, *red*: unfavorable, *green*: favorable), highlighting site 1 (the bigger sphere with no corresponding pyramid). The two nanobodies structures are overlaid and show the binding interface at complexation (59H10, *light blue*; 37E7, *purple*; p24, *dark blue*). For 59H10, the circled hydration sites are unstable and would be displaced by the binding of the p24 (sites 1 and 2) that arise in 59H10 but not 37E7 (there are no equivalent hydration sites as there are at other points in between the nanobody-antigen interface), and this approximates the difference in binding affinity. Releasing hydration site 1 (–TΔS = 1.27 kcal/mol) and 2 (–TΔS = 1.4 kcal/mol) upon complexation would expend approximately 2.67 kcal/mol –TdS, (Table S1), indicating the difference in this entropically driven interaction. *ii*) An alternative view, which shows site 1 in 59H10 would be gated by the hydrophobic F55 nearby with the unstable water displaced upon antigen binding, and this hydrophobic area protected by F55 sealing the interface. This F55 is replaced by G55 for 37E7. (*b*) Holo 59H10 and 37E7 (antigen bound). *i*) Comparison of water distributions around the binding site when the nanobodies 59H10 (*light blue*) and 37E7 (*purple*) are bound to p24 (*dark blue*). Shown are hydration sites of magnitude ΔG > |2| kcal/mol and 6 Å from the nanobody chain B for 59H10 (F55-D56-P57) in spheres and 37E7 (G55-Y56-A57) in pyramids (colored in relative, *red*: unfavorable, *green*: favorable), highlighting in particular site 1’. Site 1’ (*circled*) in the 37E7-p24 complex resides where the F55 in 59H10 is situated. *ii*) Depiction of the nanobody-p24 complex; note that the PPI interface is largely free of water with the main differences in hydration sites seen around the variable areas at the edge of the interface. *iii*) Depiction of F55 from a different viewpoint to permit comparison of the free energy density map for 59H10 F55-D56-P57 (*solid*) versus 37E7 G55-Y56-A57 (*mesh*). To see this figure in color, go online.

Fig. 6*a* shows results for WaterMaps for 59H10 and 37E7, which are more subtle to interpret. Shown are the hydration sites for 59H10 (spheres) and 37E7 (pyramids) for each nanobody, with p24 superimposed for reference at the binding interface. These are *apo* nanobody calculations of the nanobody in isolation interacting with water at its binding surface (although no p24 is bound in the calculation, it is shown in the figure). Fig. 6 *a* sections *i* and *ii* show two different viewpoints for this interaction, with p24 included to give context for the protein-protein binding interface. Again, only sites with ΔG > |2| kcal mol^−1^ are indicated for clarity and for those sites that are 6 Å from the chain B (nanobody). It can be seen that most hydration sites overlay and are equivalent, except for the site labeled 1, which indicates a hydration site on the 59H10 binding face that is unstable relative to bulk water and will favorably be displaced upon binding of p24. The equivalent site is missing in 37E7. In particular, site 1 resides at the 59H10-p24 interface and is gated, as it were, by F55 (blue) of the triplet F55-D56-P57, which in 37E7 is the much smaller glycine (G55-Y56-A57, purple). The hydrophobic Y56 of 37E7 protrudes out toward the bulk solvent, in contrast to the hydrophobic and much less soluble F55 that “seals” the interface between proteins upon binding. Displacement of water site 1 upon binding of the two hydrophobic faces on the nanobody and p24 would liberate approximately ΔG = 2.26 kcal mol^−1^(or 9.46 kJ mol^−1^, Table in Fig. 6 *a*), which compares to the ΔΔG relative difference between 59H10 and 37E7 with the F55G-D56Y-P57A mutation (Table 3) of 3.08 ± 0.71 kcal mol^−1^ (12.9 ± 3.0 kJ mol^−1^). The G55-Y56-A57 combination in 37E7 does not similarly protect this hydrophobic interface, which likely accounts for diminished binding. Fig. 6 *b* shows a WaterMap for the *holo* nanobody (p24-bound form used in the calculation and shown in the figure). A comparison is shown of the hydration sites for 59H10 (spheres) and 37E7 (pyramids) for each nanobody bound to p24. These holo nanobody calculations facilitate comparison of the behavior of water around the two nanobodies when they each bind to p24, observing the water interactions surrounding the binding interface and p24 antigen. These maps show that indeed water site 1 (from Fig. 6 *a i* and *ii*) is removed by p24 binding, and that a new site 1′ appears upon 37E7 binding to p24. The free energy density for the 37E7 holo structure is shown in Fig. 6 *b iii*, and notably, the energy density clearly resides at site 1′ where F55 is placed in 59H10. This site 1′ corresponds to a ΔG of 3.2 kcal mol^−1^(13.38 kJ mol^−1^) energy cost and demonstrates that when F55 (59H10) is replaced by G55 (37E7), this large, gating hydrophobic amino acid is switched to a small one, allowing an unstable water to reside at this site that is much more unfavorable than having a phenylalanine at this position gating the interface and not permitting unstable waters to occupy this space. Deductions from these WaterMaps agree with the FEP+ calculations in Table 3, which predict a ΔΔG = 3.75 ± 0.52 kcal mol^−1^ (15.7 ± 2.2 kJ mol^−1^) upon the mutation F55G. Table 3 lists the ΔΔG effect on affinity by stepwise mutation from 59H10 to 48G11 and 37E7, with combinations of mutations broken down into their contributory effects. Although the WaterMap and FEP+ are in good agreement, they do substantially differ from the experimentally observed value of ΔΔG = −1.6 kcal mol^−1^, i.e., the difference between ΔG 59H10 and ΔG 37E7 in Table 1. Although convergence was successfully reached in the calculations, if this research were to be extended, perhaps targeting different areas for the REST region in the FEP+ would be appropriate and investigating how the assay conditions used in the experiment (salt strength and buffer type) could have contributed to this mismatch. This analysis is useful with respect to determining how each step, and combination of steps, results in different affinities. In the case of 37E7, for example, it can be seen that the D56Y mutation is marginally (but not significantly) favorable, that P57A is not favorable (but the effect is small compared with F55G), and F55G is clearly largely accountable for the diminished binding observed for 37E7 in agreement with observations from the WaterMap. These results can be intuited, but also more rigorously accounted for both qualitatively and quantitatively with the combination of WaterMap results and FEP+ simulation protocols described here.

These WaterMap analyses accurately predict a selectivity difference exemplified by the high binding affinity of 59H10 compared with the moderate and low binding affinities of 37E7 and 48G11 respectively. The FEP+ results concur, and we conclude that a combination of these tools help to understand the affinity of nanobodies to p24 antigen much more accurately and easily than MD alone. Data from MD in isolation would be insufficient to explain the interaction based solely on interprotein hydrogen bonds and van der Waals interactions.

## Discussion

This paper reports on the biophysical characterization of a high-affinity nanobody, 59H10, a closely related variant from the same llama library (37E7), and another generated by site-directed mutagenesis (48G11). Our laboratory and others have used 59H10 as a central component of novel diagnostics for early detection of HIV (10,11), and 37E7 has been used to explore capsid polymerization mechanisms and cofactor-capsid interactions (27,28). Previously we reported the crystal structure of 59H10 and the antigen p24, and we used MD based on these structural data to model putative interactions of all three nanobodies with p24. Using these simulations, we hypothesized whereupon the differences in affinity between 59H10 and 37E7 or 48G11 might arise. In this study, we bolster and broaden our previous in silico molecular modeling simulations with biophysical characterization methods (CD and ITC) to validate our hypotheses, and we use WaterMap and FEP+ to explore the influence of interactions between water molecules and hydrophobic residues on thermodynamic parameters of binding.

Using CD spectroscopy, we show that all three nanobodies have, as expected, spectra dominated by antiparallel β-sheets. 37E7 and 48G11 have a higher underlying level of disorder without any positive signal at 222 nm (Fig. 2 *a*); this should not be ruled out as a potential contributing factor toward the less entropically driven interactions observed for 37E7 and 48G11 in contrast to 59H10. MD analysis of 1000 ns for the nanobodies in both isolation and complex (Fig. 2 *b* and *d* respectively) demonstrate that residues G54 and D97 at the top edges of the protein-protein interface fluctuate less upon binding. The standard view of antibody-antigen interactions, based on conventional antibodies, is that contact between the two is mediated mostly by the CDR3 loop. Based on this, it has previously been hypothesized that nanobodies with extended CDR3 loops (the “key”) could bind to their targets through access to antigen clefts (the “lock”) inaccessible to larger conventional antibodies (29). For the nanobodies described here, the CDR3 loops are comparably shorter, and there appears to be little large-scale conformational change upon binding. Such a lock and key mechanism of interaction is not supported either by the MD findings. Our results support a model in which the nanobody-p24 interface is large and flat, encompassing residues that make multiple contacts across an entire façade including contributions from CDR1, CDR2, and β-sheets (about half of the total protein residues on one façade). Changes in residues distant from points of contact (for example, F55G-D56Y-P57A, at the edge of the binding interface) have a cascade effect on bonds and charges, and they impact binding in nonintuitive ways that MD simulations can assist in disentangling (Fig. S6).

In order to understand the driving forces of the high-affinity binding of 59H10 to p24 considered herein, experimental measurement of the enthalpic and entropic contributions to a reaction is required. To this end, we performed ITC with all three nanobodies and p24. These experiments showed that, as expected, 48G11 has no detectable enthalpic change upon interaction with p24 at the concentrations investigated. Despite the difference in affinity, the interactions of 59H10 and 37E7 with p24 involve a similar change in enthalpy. This means that the enthalpic component of nonbonded interactions, including formations and disruptions between 59H10 and p24, and 37E7 and p24, and between all proteins and the solvent, are approximately equivalent overall. The difference in affinity, therefore, is attributed largely to the entropic contribution. Differences in entropy could arise from the movement of water or solvent ions during binding, due to bond formation, or the interaction of hydrophobic patches. Changes in entropy can also arise from gross conformational changes on binding (including rotation) that increase or decrease the solvent-exposed surface area of the protein. However, these are unlikely to be significant contributors here as there are no large-scale changes in conformation for either 59H10 or p24 upon binding seen in the CD or MD (Fig. 2). The absolute values of ΔH and TΔS reported here are commensurate with those found previously for similar protein-protein interactions (30), though 59H10 has a comparably high entropic benefit to binding p24, –TΔS = −8.6 kcal mol^−1^ (−35.8 kJ mol^−1^) of a total ΔG = −12.5 kcal mol^−1^ (−52.1 kJ mol^−1^). This is not unusual for nanobody binding interactions, and we report here a way in which to further quantify and understand the affinity that would be desirable for many diagnostic and therapeutic applications, using the WaterMap and FEP+ protocols described.

Beginning with our previous work and expanded in this study, we have sought to understand the high-affinity binding of a nanobody to its cognate target, and how the modification of residues in CDR2 and CDR3 can comprehensively affect binding. We have accomplished this using a combination of parallel in vitro and in silico methods. 59H10 and 37E7 are useful as tools in diagnostics and exploration of viral protein chemistry (10,11,27,28). These nanobodies exhibit the same stability seen in previous studies of nanobodies under extreme conditions (10,31). The small size of nanobodies makes them easier to work with than conventional antibodies, both in vitro and in silico. The insights into the role of water molecules in mediating the high-affinity interaction of 59H10 with p24 gained from this study will be of use to those seeking to harness the potent power of nanobodies for therapeutic as well as diagnostic approaches. Understanding such binding mechanisms, pinpointing residues that can make or break a therapeutic or diagnostic, and demonstrating potential for developability have wide-reaching importance in the current climate (32).

## Author contributions

Designed research: J.B., E.G., and R.M.; performed experiments: J.B., E.G., M.G., C.L., and A.B.; analyzed data: J.B., E.G., M.G., B.M., C.L., A.B., and R.M.; drafted manuscript: J.B. and E.G.; revised manuscript: J.B., E.G., A.B., C.L., M.G., and R.M.

## Supporting citations

Reference (40) appear in the supporting material.

## Supporting material

Document S1. Figures S1–S6 and Table S1

Document S2. Article plus supporting material
