# Supplementary material for: Thermodynamic analysis of an entropically driven, high-affinity nanobody-HIV p24 interaction
Source: Biophys J. 2022 Dec 16;122(2):279–89. doi: 10.1016/j.bpj.2022.12.019 (PMC9892613; doi:10.1016/j.bpj.2022.12.019)
Supplement: Document S1. Figures S1–S6 and Table S1 [file mmc1.pdf]

Biophysical Journal, Volume *122*

Supplemental information

Thermodynamic analysis of an entropically driven, high-affinity nano-body-HIV p24 interaction

Jennifer C. Brookes, Eleanor R. Gray, Colleen N. Loynachan, Michelle J. Gut, Benjamin S. Miller, Alex P.S. Brogan, and Rachel A. McKendry

Thermodynamic, biophysical and structural insights of a nanobody HIV p24 complex

JC Brookes

ER Gray

C Loynachan

M Gut

BS Miller

APS Brogan

RA McKendry

Supplementary Methods

Biolayer Interferometry: BLI was performed using a Fortebio Octet RED96 and the data analysed essentially using the method described previously (9, 40). Briefly, p24 was immobilised onto AR2G (amine-reactive) probes using fresh sulfo-NHS-EDC, before subsequent quenching with 1M ethanolamine. Probes were equilibrated in protein storage buffer, before being dipped in seven different concentrations of nanobody for a 60 minute binding phase (the eighth probe was dipped in a fresh well of storage buffer and was used to normalize the run). At 60 minutes, probes were moved to a fresh well of storage buffer for a 60 minute dissociation phase.

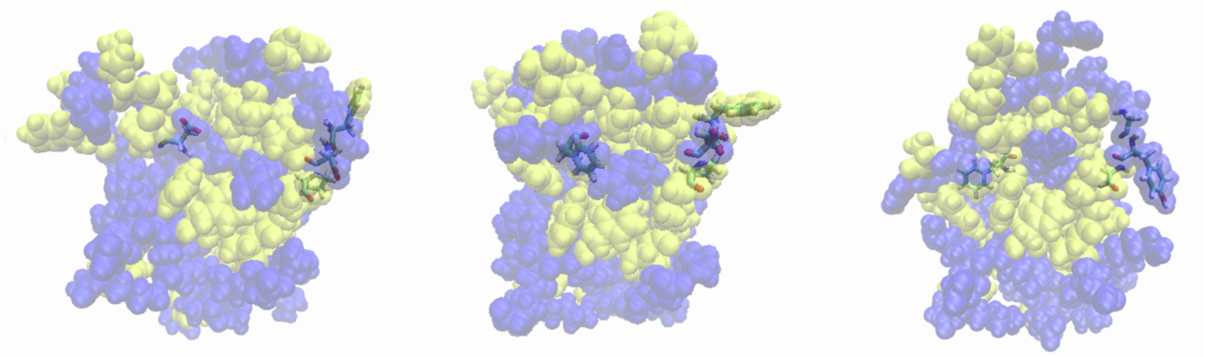

Supplementary Figure 1. Structures of the three nanobodies. Alternative view to Figure 1b, with amino acids shown in van der Waals rendering and colored by hydrophobicity (yellow - hydrophobic, blue - not hydrophobic). To highlight the differences between them, the variant amino acids in CDR2 and CDR3 are rendered in licorice.

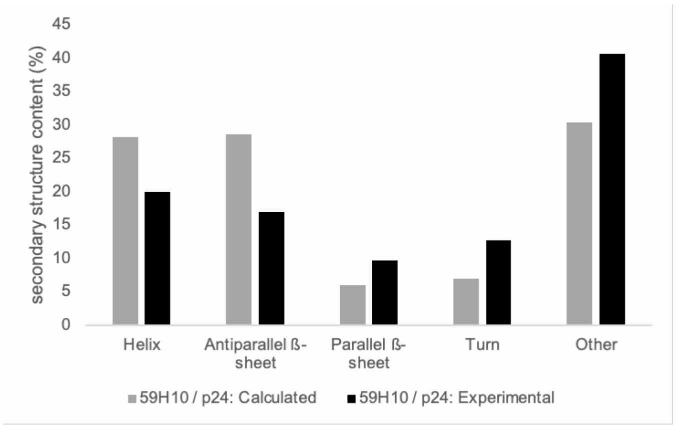

Supplementary Figure 2. Full BeStSel analysis of calculated and experimental CD data from the 59H10-p24 complex. Shown are the proportion of features from the CD data that can be classified as different types of secondary structures. The 'other' group includes 3-10 helices, S-bends, π-helices, ß-bridges and irregular loops.

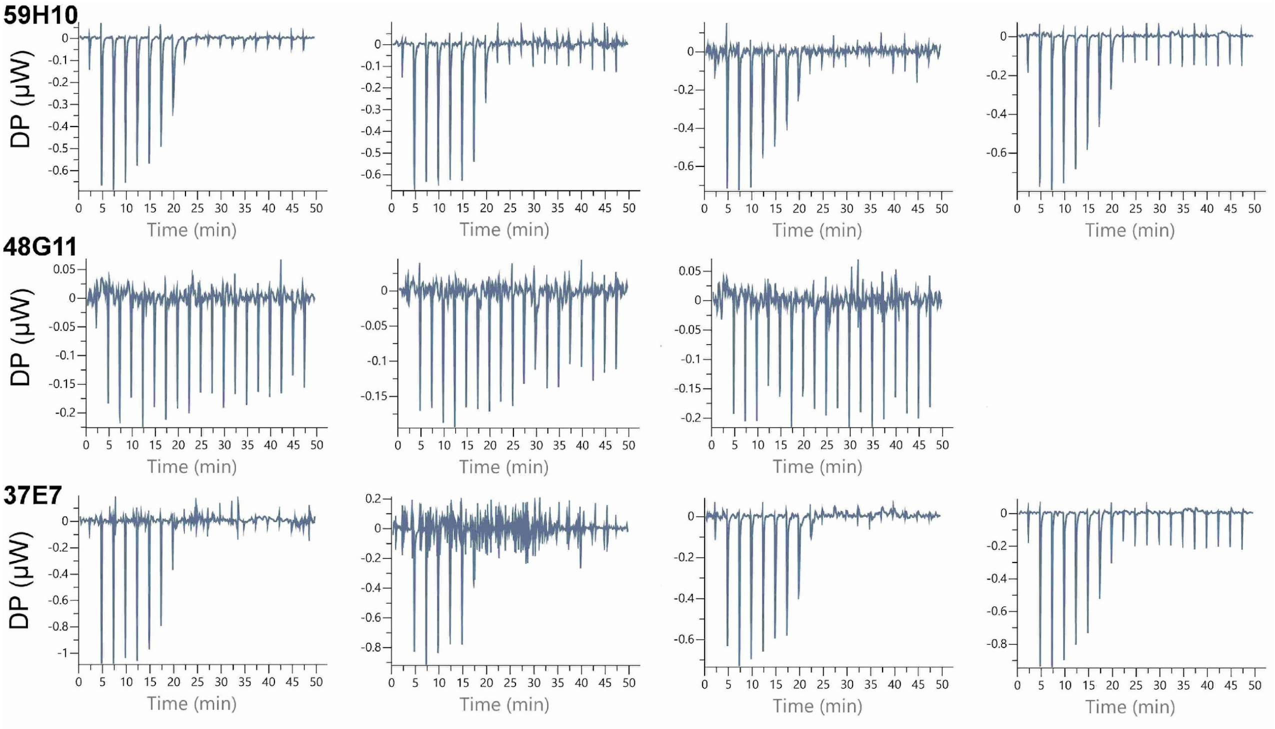

Supplementary Figure 3. Thermograms of data shown in Figure 3. Shown are the changes in differential power as 200μM nanobody in the syringe is titrated into 12.5μM p24 in the sample cell, all diluted into 10mM phosphate pH 8 with 25mM NaCl. The results of three (48G11) or four (59H10 and 37E7) independent experiments are shown.

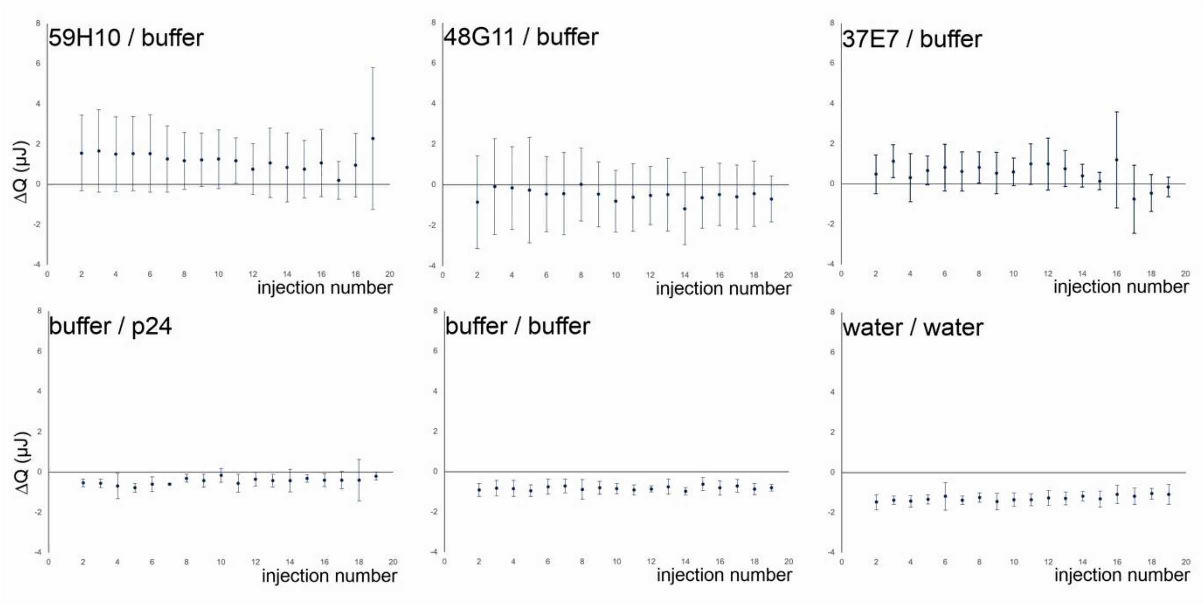

Supplementary Figure 4. Control experiments for isothermal titration calorimetry. To assess the heat of dilution of injections, nanobodies were titrated into buffer, buffer into p24 and buffer into buffer. Water was titrated into water at the beginning of all experiments. Shown are the combined results from at least 3 experiments per condition. ΔQ, change in heat.

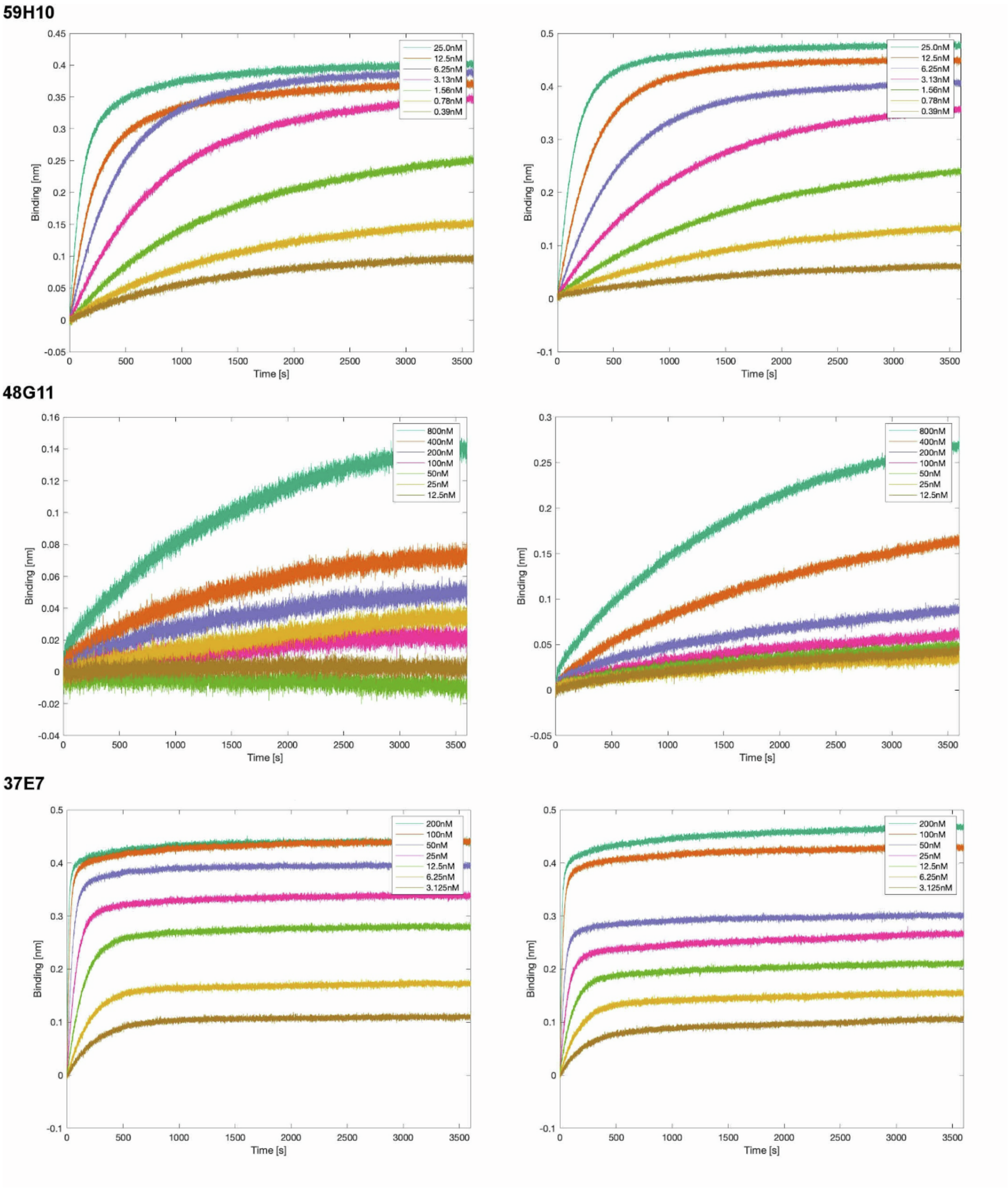

Supplementary Figure 5. Biolayer interferometry plots of nanobodies binding to p24, normalised to reference baseline. Binding was assayed in the same buffer as ITC (Figure 3 and Table 1, 10mM phosphate pH 8 with 25mM NaCl). The results of two independent experiments are shown. Definitive K_Ds were calculated using the average taken from three different methods: 1. The fitted equilibrium method described in (9); 2. FortéBio software, curve fitting method; 3. FortéBio software, steady-state analysis method.

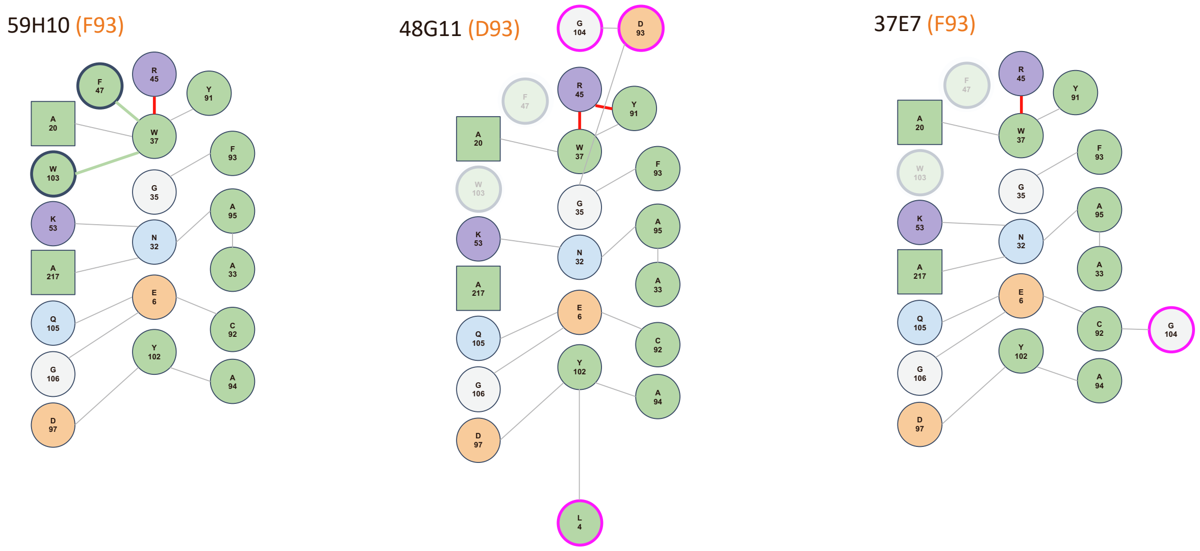

Supplementary Figure 6. Analysis of interactions for nanobody residues during 100 ns molecular dynamics simulation. Nodes are amino acids and lines connecting them demonstrate interactions and are colored by: grey, H-bond; red, pi-cation; green, hydrophobic. Residues from VHH are shown in circles, p24 in squares. Hydrophobic interactions (green) seen for 59H10 (left, F93) are lost in 48G11 (D93) and 37E7 (F93). There are internal hydrophobic contacts in 59H10 between W103, F47 to W37, suggesting these hydrophobic effects may additionally be important for high affinity binding. Residues that disappear from bond maps from 59H10 to 48G11, or 59H10 to 37E7 are highlighted with a bold border. Residues that appear on bond maps for 48G11 or 37E7 (but not 59H10) are drawn on the periphery with a pink border.

Site	Occupancy	Overlap	ΔH kcal/mol	-TΔS kcal/mol	ΔG kcal/mol	Hydrogen bonds (water to water)	Hydrogen bonds (protein to water)
1 (apo)	0.46	1.00	0.99	1.27	2.262	2.09	0.00
1' (holo)	0.39	0.00	2.04	1.16	3.20	1.35	0.93

Supplementary Table 1. Quantitative comparison of the hydration site marked in Figure 6 for apo and holo forms of the nanobody. See Figure 6 for the marking of 1 and 1' within the context of the nanobody-p24 complex.
